# Door-to-Needle Optimization in Acute Ischemic Stroke: A Review of Rapid Emergency Department Interventions

**DOI:** 10.26502/fccm.92920503

**Published:** 2026-09-06

**Authors:** Fihr Chaudhary, Nasr Chaudhary, Sahil Afzal, Devendra K. Agrawal

**Affiliations:** 1 Department of Translational Research1, College of Osteopathic Medicine of the Pacific,Western University of Health Sciences, Pomona, California 91766 USA; 2 The San Joaquin Valley PRIME+ BS-MD Program, University of California, Merced, CA 95343, USA.; 3 University of California, Merced, CA 95343, USA

**Keywords:** Acute ischemic stroke, Alteplase, Door-to-needle time, Emergency department workflow, Endovascular thrombectomy, Helsinki model, Intravenous thrombolysis, Prehospital stroke care, Quality improvement, Tenecteplase

## Abstract

Acute ischemic stroke is the second leading cause of death worldwide, and the benefit of intravenous thrombolysis decays steeply with time while its hemorrhagic risk does not. That asymmetry makes the door-to-needle interval, measured from emergency department arrival to administration of the thrombolytic bolus, one of the few determinants of stroke outcome that can be improved without new technology or additional resources. This review synthesizes the evidence underlying door-to-needle optimization. It begins with the quantified biology of delay, in which roughly 1.9 million neurons are lost for every minute of untreated large-vessel ischemia, and with the pooled randomized data showing that the number needed to treat for one additional good outcome rises from approximately five within ninety minutes to approximately fifteen at the outer edge of the 4.5-hour window. It then examines how the interval should be measured, arguing that the mean and the minimum door-to-needle time must both be reported because they describe different properties of the same system. Prehospital determinants of delay, including field triage instruments, dispatcher and paramedic education, prenotification, and ambulance-based thrombolysis, are reviewed alongside the in-hospital Helsinki protocol, whose parallelization of imaging, clinical assessment, and drug preparation reduced median in-hospital delay to twenty minutes and was reproduced in Melbourne within four months. National registry data from the Target: Stroke initiative confirm that these gains scale across a hospital network with concurrent reductions in mortality and symptomatic hemorrhage. The review closes with the trade-off between speed and diagnostic certainty, the reassuring safety profile of thrombolysis in stroke mimics, and the way tenecteplase, imaging-based patient selection, and endovascular thrombectomy are reshaping what the metric measures.

## Introduction

Worldwide, around 11% of all fatalities are caused by stroke, making it the second leading cause of death. Over the past eight decades, stroke has slipped from the position of second to fourth major cause of death in the US [1–9]. In particular, the death rate following a stroke has declined within the past two decades [10]. Enhanced prevention and care were two of many contributing factors to this accomplishment [11]. Patients suffering from stroke greatly benefit from being started on thrombolytic medication as soon as possible [12–16].

The success of tissue plasminogen activator (t-PA) is time-dependent since brain tissue is very sensitive to ischemia. Consequently, as time grows, the number needed to treat (NNT) for a single patient to recover also increases (<1.5 h NNT=5; 1.5–3 h NNT=9; 3–4.5 h NNT=15) [17]. That is why it is so important to start a good thrombolytic treatment as soon as possible when symptoms appear. This duration may be broken down into two parts: the first is the time it takes from symptom onset until someone reaches the emergency room, and the second is the time it takes for thrombolytic therapy to be administered once someone arrives at the hospital, conventionally called the door-to-needle time. It is highly recommended that a uniform approach be established for the care of stroke to optimize the door-to-needle time [11]. Effectiveness is typically evaluated by calculating the mean door-to-needle time over several patients. The peak performance, also known as the minimal door-to-needle time, is another statistic that could be considered valuable in determining the effectiveness of a stroke management system that has been deployed.

This review critically examines the evidence underlying door-to-needle optimization in acute ischemic stroke. It addresses why treatment delay carries the weight that it does, how the interval is defined and measured, which prehospital and in-hospital factors account for most of the delay, what structured protocols such as the Helsinki model and the Target: Stroke initiative have achieved, and where the trade-off between speed and diagnostic certainty lies. It closes by considering how the door-to-needle metric is changing as tenecteplase, imaging-based patient selection, and endovascular thrombectomy alter the shape of the acute stroke pathway.

### The Time Dependence of Reperfusion

The rationale for compressing the door-to-needle interval rests on a well-characterized biological relationship. In the typical large vessel supratentorial ischemic stroke, an estimated 1.9 million neurons, 14 billion synapses, and 12 kilometers of myelinated fibers are lost for each minute that reperfusion is delayed, and the ischemic brain ages roughly 3.6 years for each hour without treatment [18]. This quantification converts an abstract urgency into a rate, and it is the reason that time savings measured in single minutes are considered clinically meaningful in this disease and in few others. The clinical evidence matches the biology. A pooled analysis of individual patient data from nine randomized trials of alteplase against placebo or open control, comprising 6,756 patients, demonstrated that the proportion of patients achieving a good stroke outcome declined steadily as the onset-to-treatment interval lengthened, with the odds ratio for a favorable outcome falling from approximately 1.75 when treatment was given within three hours to approximately 1.26 in the three-to-4.5-hour window [19]. The same analysis showed that the relative increase in fatal intracranial hemorrhage attributable to alteplase was similar regardless of treatment delay, meaning that the harm is fixed while the benefit decays. That asymmetry is what makes delay costly rather than merely suboptimal.

Earlier pooled work had already expressed this relationship in terms directly usable at the bedside, showing that the number needed to treat rises from roughly five when thrombolysis is delivered within ninety minutes to roughly fifteen at the outer edge of the window (Figure 1) [17]. In-hospital mortality falls by approximately five percent for every fifteen minutes by which the door-to-needle time is reduced [12]. The benefit is not confined to the acute hospitalization. In a retrospective cohort of Medicare beneficiaries treated with intravenous t-PA, every fifteen-minute reduction in door-to-needle time was associated with lower one-year all-cause mortality and lower one-year all-cause readmission [20]. Speed, in other words, buys durable outcomes rather than only better discharge examinations.

### Defining and Measuring the Door-to-Needle Interval

Until recently, the official aim for the treatment of ischemic stroke has been to achieve a door-to-needle time of sixty minutes [3,9]. Current guidance retains the sixty-minute benchmark as a floor for most eligible patients while establishing a more demanding secondary target of forty-five minutes in a substantial proportion, reflecting the demonstrated feasibility of shorter intervals [21]. How the interval is measured deserves scrutiny, because the mean and the minimum answer different questions. The mean door-to-needle time across a cohort describes typical system performance and is the metric on which institutions are benchmarked. The minimum, or peak performance, describes what the system is capable of when every element functions as designed. The two can diverge sharply, and many factors unrelated to the quality of the stroke pathway itself will adversely affect the mean. An institution that uses magnetic resonance imaging to select patients with an ambiguous time of symptom onset, for example, will record a longer mean door-to-needle time than an institution without access to magnetic resonance imaging for this indication, even though the former is delivering more appropriate care to a broader population. For this reason both statistics should be reported when a local protocol is being evaluated, since the minimum reveals whether a protocol works under ideal conditions while the mean reveals how often ideal conditions obtain.

The magnitude of the opportunity has been well quantified. Historically, only 26.6% of patients who arrived in time to receive t-PA were treated within an hour of arrival [22]. A nationwide analysis of 154,221 patients receiving t-PA within three hours of onset across 913 hospitals found that median door-to-needle times fell from 78 minutes before any structured intervention to 66 minutes and then to 50 minutes across two successive phases of a quality improvement program, while the proportion treated within sixty minutes rose from 26.4% to 42.7% and then to 68.6% [23]. The magnitude of that shift indicates that most of the delay was never an unavoidable property of stroke care, but a property of how the pathway was organized.

### Prehospital Determinants of Delay

Roughly half of the interval between symptom onset and treatment occurs before the patient reaches the hospital, and improvements in stroke management are required both before hospitalization and once the patient has arrived [24].

After arriving at the patient's location, emergency medical services personnel conduct a brief clinical assessment, which typically includes the Face Arm Speech Test, to identify patients who may be suffering from a stroke [25,26]. Instruments of this kind form the foundation of the prehospital pathway, and their diagnostic performance and limitations in the emergency setting have been reviewed in detail [27]. If the clinical signs point to the possibility of a stroke, the patient must be taken to the emergency room without unnecessary delay. If it is at all possible, the paramedics will obtain a medication plan for the patient, together with the telephone numbers of the family physician and any nearby relatives. This information is often the rate-limiting step in the thrombolysis decision and collecting it in the field removes that step from the in-hospital interval entirely. To improve the ability of rescue services to identify strokes and administer adequate prehospital stroke care, it is widely recommended that paramedics participate in education programs led by experienced neurologists [28]. Dispatchers should be included in these programs as well, so that they can improve their ability to identify the signs and symptoms of stroke during the initial call [29]. Prenotification is the other high-yield prehospital intervention. Door-to-needle time improves when emergency medical services notify hospitals in advance, when computed tomography is quickly available, when t-PA is stored and administered in the emergency department rather than dispensed from a central pharmacy, and when an in-house stroke expert is available at all times [30]. Whether appropriately trained paramedics require an accompanying emergency medical services physician, and whether the addition of a physician introduces delay at the point of dispatch, remains unresolved. The physician arrival adds an interval on scene, but the information gathered during that interval, including the medication list, contact numbers, and relevant medical history, is precisely what permits thrombolysis to proceed without waiting for laboratory results after arrival. Whether suitably educated paramedics can perform the same information-gathering function without physician support has not been formally established.

The most aggressive prehospital strategy moves the treatment to the patient rather than the reverse. Ambulance-based thrombolysis, delivered from a vehicle equipped with a computed tomography scanner and point-of-care laboratory testing, was shown to substantially reduce time to thrombolysis in a randomized comparison against conventional care [24]. A subsequent prospective multicenter controlled trial found that patients managed by mobile stroke units had better utility-weighted disability outcomes at ninety days than those managed by conventional emergency medical services, with the benefit driven largely by a higher proportion treated within the first hour after onset [31]. Mobile stroke units are resource-intensive and are not a realistic option for most systems, but their results establish an upper bound on what prehospital optimization can achieve and confirm that the time saved translates into preserved function.

### In-Hospital Determinants: The Helsinki Model

The best-documented in-hospital protocol originates from Helsinki, where a series of sequential process changes reduced the median in-hospital delay to twenty minutes [32]. The protocol is not a single intervention, but a bundle of roughly a dozen measures, most of which involve moving a step earlier in the sequence, performing steps in parallel rather than serially, or eliminating a wait that does not change the treatment decision.

The core elements are as follows. The emergency department receives a telephone call informing them of the impending arrival of a suspected stroke, together with information regarding the time at which symptoms first appeared. It is then the responsibility of the physician overseeing the emergency department to contact the neurologist, who is required to be present in the emergency department before the arrival of the patient. The emergency physician prepares for full monitoring, including continuous intraarterial blood pressure management and airway management, if these are required. The computed tomography scanner is blocked for routine examinations. Emergency medical services will, where possible, obtain blood samples during transport and forward them on arrival. To begin thrombolysis there is no need to wait for the values of the international normalized ratio and platelet count if a medication plan and information about the patient's past medical history are available, since the pretest probability of a clinically relevant coagulopathy in a patient on no anticoagulant is very low.

As soon as the patient arrives, they are moved directly from the ambulance stretcher onto the computed tomography table rather than onto an emergency department bed. Emergency medical services personnel provide their handover to the neurologist and the emergency physician simultaneously. Nurses attend to monitoring equipment, supplemental oxygen, body temperature, and venous access during the transfer. The neurologist's evaluation is deliberately narrow: determining the National Institutes of Health Stroke Scale score and taking a brief medical history restricted to information pertinent to the thrombolysis decision. While this is occurring, the emergency physician examines the patient with particular attention to airway, breathing, and circulation, and places an intraarterial line for continuous pressure monitoring. The insertion of that catheter must not postpone the commencement of imaging, and if it is not successfully placed after two attempts the procedure is abandoned. While the scan is being performed, the neurologist confirms that all information required for the thrombolysis decision is available apart from the imaging result itself, and gathers any missing information, for example by telephoning relatives or the family physician.

The most distinctive element is the premixing of t-PA before the scan is performed, in cases where the clinical picture strongly suggests an ischemic rather than a hemorrhagic origin. Because the drug has already been reconstituted in the computed tomography room, it can be administered immediately once the non-contrast study excludes hemorrhage. While the emergency physician administers the bolus, the nurse prepares the infusion that will follow. Additional computed tomography angiography and perfusion studies are carried out at this point if they are indicated, after the bolus rather than before it, so that vascular imaging never sits on the critical path to treatment. Figure 2 contrasts this parallel arrangement with the conventional serial sequence. The transferability of this protocol has been formally tested. Implementation at a hospital in Melbourne reduced the median in-hours door-to-needle time from 43 minutes to 25 minutes within four months and did so in the absence of a dedicated neurological emergency department and without electronic patient records, both of which are features of the Finnish system [33]. That result matters because it addresses the most common objection to the Helsinki data, namely that the result depends on structural advantages that other systems cannot replicate.

The most compressed door-to-needle times described in the literature, on the order of seven minutes, have been achieved under conditions in which every element of this protocol functioned simultaneously, and the diagnosis was clinically unambiguous [12]. Such intervals cannot be recommended as a target, since they depend on a favorable alignment of circumstances that cannot be engineered on demand, and pursuing them as a routine objective would inevitably trade diagnostic accuracy for speed. What they do establish is the lower bound of what a well-designed system can achieve, and they permit any institution to evaluate its own protocol against a benchmark measured under ideal conditions.

### Nationwide Quality Improvement

Whether the gains demonstrated in individual academic centers can be reproduced at scale was addressed by the Target: Stroke initiative, a national quality improvement program organized around a defined set of best-practice strategies. The first phase, evaluated across 71,169 patients at 1,030 hospitals, was associated with a reduction in median door-to-needle time from 77 to 67 minutes and an increase in the proportion of patients treated within sixty minutes from 26.5% to 41.3%, accompanied by reductions in in-hospital mortality and symptomatic intracranial hemorrhage [34]. The improvement in outcomes alongside the improvement in process is the important observation, since it excludes the possibility that faster treatment was achieved by treating a lower-risk population. The second phase extended these gains further, with median door-to-needle times reaching 50 minutes and the proportion treated within forty-five minutes rising from 10.1% before intervention to 41.4% (Figure 3) [35]. The specific strategies most strongly associated with improvement have been characterized separately and align closely with the Helsinki elements: emergency medical services prenotification, rapid triage and stroke team activation, single-call activation systems, premixing of the thrombolytic, transporting the patient directly to imaging, rapid acquisition and interpretation of brain imaging, and prompt data feedback to the treating teams [30].

What distinguishes a successful program from an unsuccessful one appears to be less the selection of any individual strategy than the number adopted and the consistency of adherence. Hospitals that implemented a larger share of the recommended strategies achieved shorter times, which suggests that the effect is cumulative and that partial implementation yields partial benefit.

### Barriers, Trade-offs, and Stroke Mimics

Several factors reliably lengthen the door-to-needle interval. A lack of reliable information regarding previous medications, particularly anticoagulants, requires either laboratory confirmation or a decision made under uncertainty. Uncontrolled arterial hypertension requires treatment before thrombolysis can proceed. An ambiguous or unknown time of symptom onset requires either exclusion from treatment or additional imaging to establish eligibility. Patients presenting with milder or atypical deficits are recognized more slowly, both prehospital and in the emergency department. The central trade-off in door-to-needle optimization is between speed and diagnostic accuracy. Compressing the interval necessarily reduces the time available to consider alternative diagnoses, and the rate of inadvertent thrombolysis in patients who turn out not to have had a stroke is the metric by which this is monitored. It is generally recommended that the proportion of thrombolytic treatments delivered to stroke mimics be kept below three percent, although a higher rate may be acceptable where the balance between treatment speed and diagnostic accuracy is deliberately and transparently managed [11].

The safety data on this point are reassuring and have shifted the calculus. A prospective five-year single-center series combined with a comprehensive meta-analysis of published case series found that the rate of symptomatic intracerebral hemorrhage among patients with stroke mimics who received intravenous thrombolysis was very low, substantially lower than among patients with confirmed ischemic stroke, and that the favorable natural history of the mimics was not adversely affected [35]. The implication is that delaying treatment to exclude a mimic imposes a definite cost on the majority who are having a stroke in exchange for avoiding a harm that is, in the mimic population, small. This does not license carelessness, but it does establish the direction in which uncertainty should be resolved.

Patients with an unknown time of onset represent a distinct problem rather than a delay to be eliminated. Approximately one in five ischemic strokes is recognized on waking, and these patients were historically excluded from thrombolysis altogether. A randomized trial demonstrated that among patients with an unknown onset time selected by a mismatch between the diffusion-weighted and fluid-attenuated inversion recovery magnetic resonance sequences, intravenous alteplase produced better functional outcomes than placebo [36]. Imaging-based selection therefore extends eligibility to a population that would otherwise receive nothing, and the additional imaging time it requires should be understood as expanding access rather than as a failure of process. This is precisely the circumstance in which the mean door-to-needle time of an institution can worsen while the quality of its care improves.

### Extending the Paradigm: New Agents and Endovascular Therapy

Two developments are reshaping what the door-to-needle metric measures. The first is the substitution of tenecteplase for alteplase. Tenecteplase is administered as a single bolus rather than as a bolus followed by a sixty-minute infusion, which removes the preparation and administration of an infusion from the critical path. A pragmatic multicenter randomized non-inferiority trial of 1,577 patients found that tenecteplase at 0.25 mg/kg was non-inferior to alteplase with respect to excellent functional outcome at ninety days, with comparable rates of symptomatic intracerebral hemorrhage and ninety-day mortality [37]. The workflow advantage is most pronounced in patients requiring interhospital transfer for thrombectomy, in whom the infusion would otherwise have to be maintained during transport.

The second development is endovascular thrombectomy for large-vessel occlusion. A meta-analysis of individual patient data from five randomized trials established that thrombectomy benefits most patients with acute ischemic stroke caused by proximal anterior circulation occlusion, irrespective of patient characteristics or geographical location [38]. Its arrival means that the emergency department pathway now has two endpoints rather than one, and that vascular imaging has become a necessary rather than an optional component of the acute workup. The Helsinki sequencing principle applies here as well: angiography is performed after the thrombolytic bolus rather than before it, so that the additional imaging required to identify thrombectomy candidates does not lengthen the interval for the larger group who will be treated with thrombolysis alone. The consequence is that door-to-needle time, while it remains the correct metric for intravenous thrombolysis, is no longer sufficient on its own to describe the performance of an acute stroke system. Door-to-imaging, door-in-door-out for transferred patients, and door-to-groin-puncture for thrombectomy candidates each measure a distinct segment of a pathway that has become branched. A system that has optimized only the thrombolysis branch may still be losing a large amount of time in the branch that leads to the angiography suite.

## Discussion

The literature reviewed here supports several conclusions that are reasonably firm. Treatment delay carries a quantified biological and clinical cost, the benefit of thrombolysis decays with time while its risk does not, and the association between shorter door-to-needle times and better outcomes extends to one-year mortality and readmission rather than being confined to short-term functional measures [19,20]. Structured protocols reduce the interval substantially, and the reduction has been reproduced both in individual centers on different continents and across a national hospital network [23,32, 33]. The strategies that produce the reduction are known and have been enumerated [30].

Several questions are less settled. The relative contribution of each individual measure within a bundle has not been isolated. Of the roughly twelve elements of the Helsinki protocol, it is not known how much time is saved by premixing the thrombolytic as opposed to, for example, transporting the patient directly to the scanner or having the neurologist present before arrival. Nor is it known whether the elements interact, such that a measure that yields little benefit in isolation becomes valuable once others are in place. Answering these questions would require factorial designs that have not been performed and that would be difficult to conduct ethically once the bundle as a whole is established as beneficial. It is also unclear how far the interval can be compressed before diagnostic accuracy is compromised in a way that matters. The stroke mimic literature suggests the safety margin is wider than was once assumed [35], but that literature comes predominantly from experienced centers with immediate neurological expertise, and its generalizability to hospitals without an in-house stroke physician is uncertain. The optimal balance is likely to differ between institutions rather than being a single number. A further limitation concerns the metrics themselves. Because the mean door-to-needle time is influenced by case mix, imaging strategy, and the proportion of patients with an unknown onset time, comparisons between institutions using this figure alone are unreliable. Reporting the minimum alongside the mean, as has been proposed, addresses part of this problem but introduces its own difficulty, since a single very short interval may reflect an unusually favorable presentation rather than a well-functioning system.

Finally, the field is moving. As tenecteplase replaces alteplase and as thrombectomy becomes available to a larger proportion of patients, the pathway that door-to-needle time was designed to measure is changing shape. Future work on acute stroke process improvement will need to account for a branched pathway with multiple endpoints rather than the single linear sequence that the original metric assumed.

## Conclusion

The evidence that rapid thrombolysis improves outcomes in acute ischemic stroke is not in dispute, and neither is the evidence that most institutions treat more slowly than they can treat. The gap between the sixty-minute guideline target and the twenty-minute intervals achieved by structured protocols is not explained by differences in resources or patient populations but by differences in how the pathway is organized: whether steps are performed in parallel or in sequence, whether waits that do not change the treatment decision have been eliminated, and whether the thrombolytic is prepared before rather than after the decision to give it. Every institution can measure its own performance against these benchmarks, and both the mean and the minimum door-to-needle time should be reported when a local protocol is evaluated, since the two describe different properties of the same system. Door-to-needle optimization remains one of the few interventions in acute stroke care that requires no new technology and yields benefit at the level of individual minutes.

## Figures and Tables

**Figure 1:**
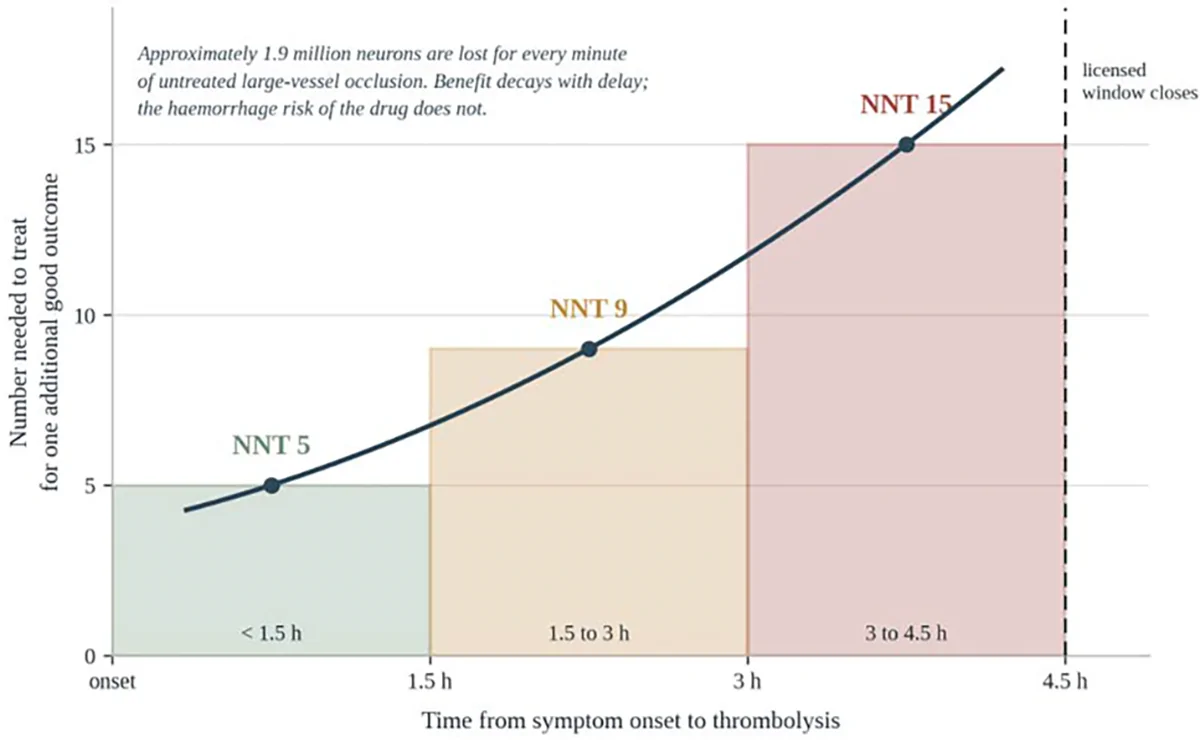
Decay of thrombolytic benefit with increasing delay from symptom onset. **Note**: Shaded blocks give the number needed to treat for one additional good outcome within each time band, plotted from values reported in the pooled trial literature; the curve is fitted through the band midpoints. Because the risk of the drug does not fall as the benefit does, delay progressively worsens the balance of the two.

**Figure 2:**
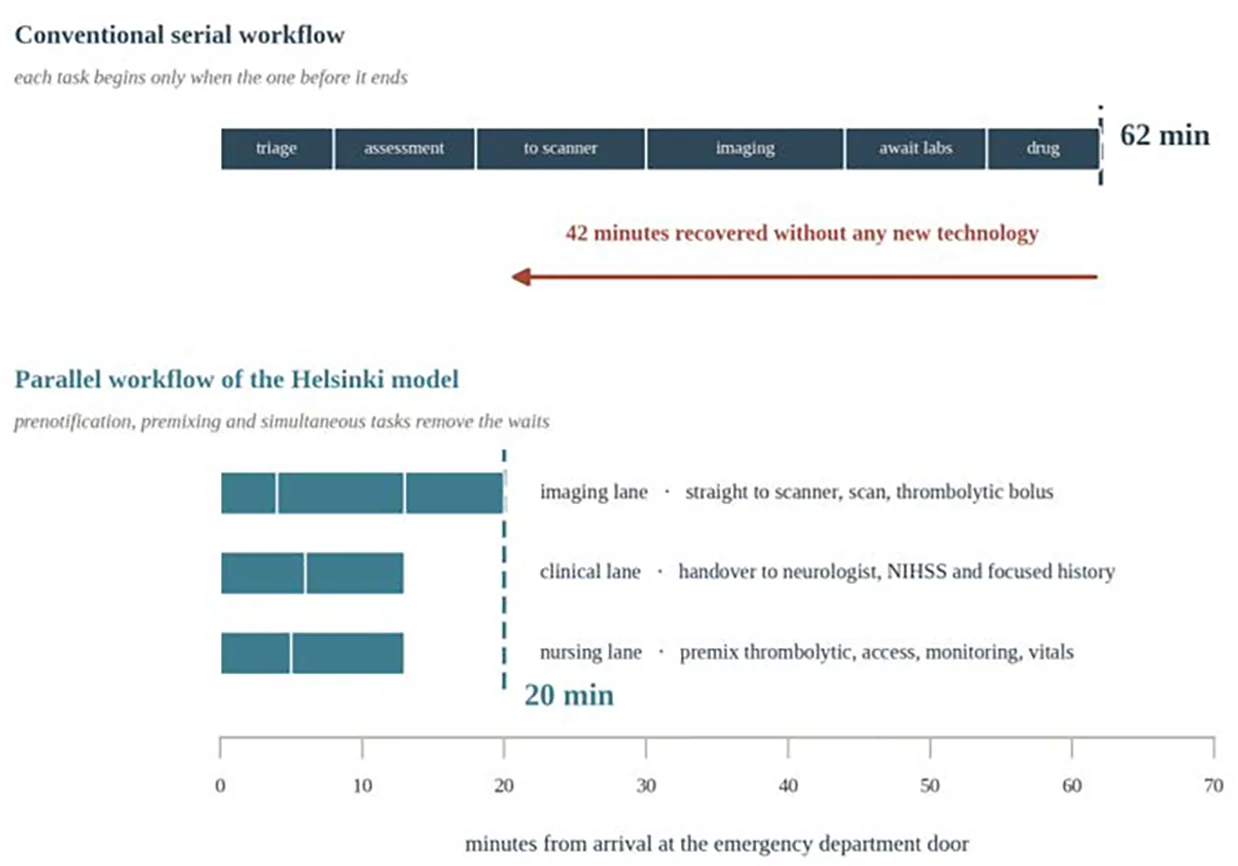
Serial and parallel arrangements of the same in-hospital tasks, drawn on a common time axis. **Note**: In the conventional arrangement each task waits for the one before it. In the parallel arrangement the imaging, clinical and nursing tasks proceed simultaneously, and the thrombolytic is reconstituted before the scan is read. Durations are illustrative and are used to show where time is recovered rather than to represent any single institution.

**Figure 3:**
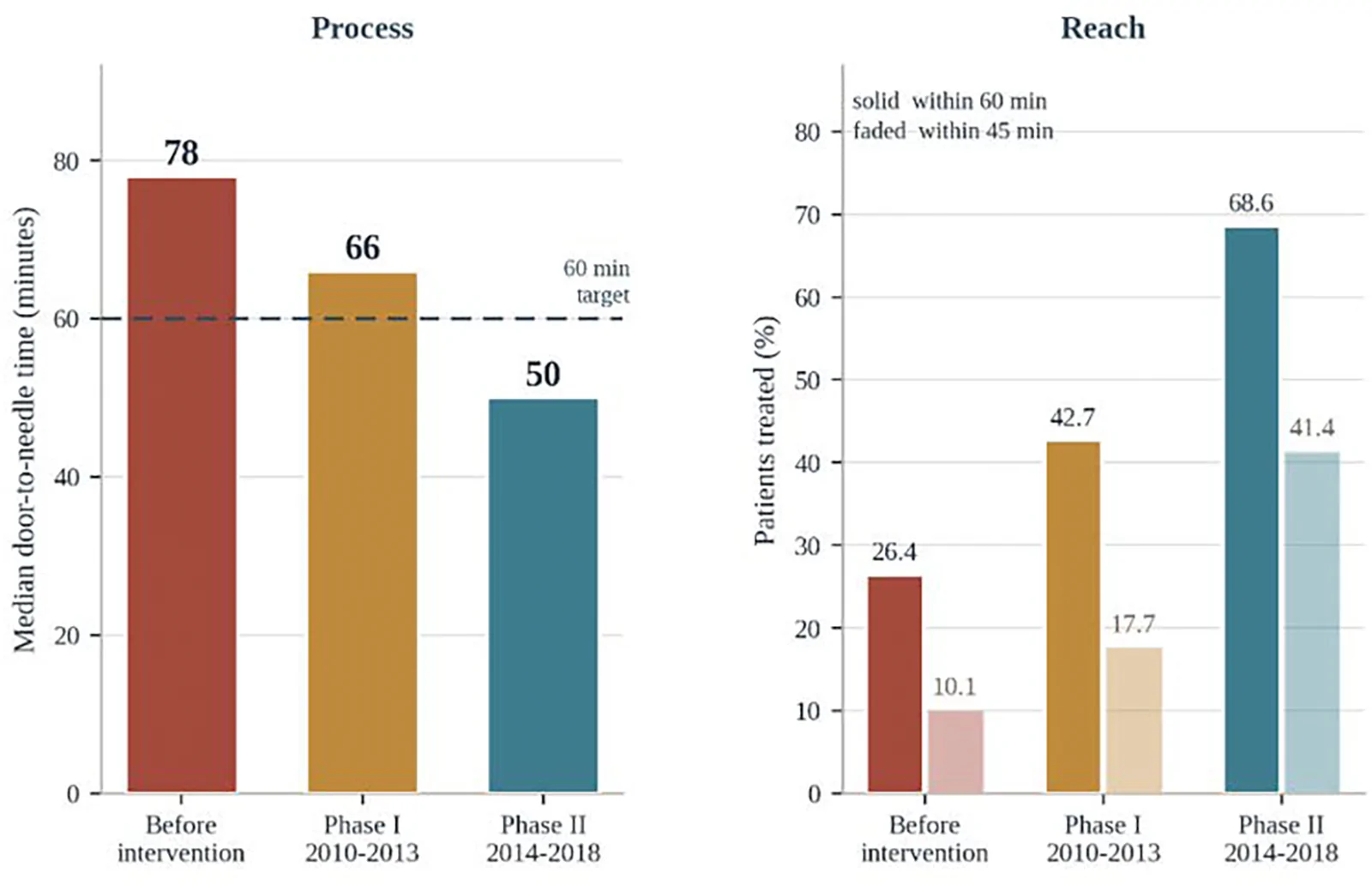
Effect of a national door-to-needle quality improvement programs on process and on reach. **Note:** The data are plotted from reported registry values across 913 hospitals. The left panel shows the fall in median door-to-needle time against the sixty-minute guideline target. The right panel shows the proportion of treated patients meeting the sixty-minute and forty-five-minute thresholds.

## Key Points

- Thrombolytic benefit decays with time while the hemorrhagic risk of the drug remains fixed, so delay progressively worsens the balance of the two rather than simply postponing an unchanged gain.
- An estimated 1.9 million neurons, 14 billion synapses, and 12 kilometers of myelinated fibers are lost for each minute of untreated large-vessel ischemia, which converts urgency into a measurable rate.
- The number needed to treat for one additional good outcome rises from roughly five when thrombolysis is delivered within ninety minutes to roughly fifteen at the outer edge of the treatment window.
- Each fifteen-minute reduction in door-to-needle time is associated with lower in-hospital mortality and with lower one-year mortality and readmission, so speed buys durable outcomes rather than only better discharge examinations.
- Current guidance retains sixty minutes as the floor for most eligible patients while adding a more demanding forty-five-minute secondary target that structured protocols have shown to be attainable.
- Both the mean and the minimum door-to-needle time should be reported when a local protocol is evaluated, since the mean is confounded by case mix and imaging strategy while the minimum reveals what the pathway achieves when every element functions as designed.
- Roughly half the onset-to-treatment interval accrues before hospital arrival, which makes EMS prenotification, dispatcher and paramedic education, and field collection of the medication list among the highest-yield targets available.
- The Helsinki bundle reduced median in-hospital delay to twenty minutes by performing tasks in parallel, moving patients directly from the ambulance stretcher onto the CT table, and reconstituting the thrombolytic before the scan is read; the same protocol cut Melbourne times from 43 to 25 minutes in four months without the structural advantages of the Finnish system.
- The Target: Stroke initiative reproduced these gains at national scale, lowering median door-to-needle time from 78 to 50 minutes with concurrent reductions in mortality and symptomatic intracranial hemorrhage, which excludes the possibility that speed was achieved by treating a lower-risk population.
- Thrombolysis of stroke mimics carries a symptomatic hemorrhage rate well below that of confirmed stroke, so residual diagnostic uncertainty should generally be resolved toward treating, while the arrival of tenecteplase and thrombectomy means door-to-needle time alone no longer describes a pathway that has become branched.
